# Caregivers Clinic at Memorial Sloan Kettering Cancer Center

**DOI:** 10.1093/geroni/igab046.1188

**Published:** 2021-12-17

**Authors:** Allison Applebaum

**Affiliations:** Memorial Sloan Kettering Cancer Center, New York, New York, United States

## Abstract

The mission of the Caregivers Clinic at Memorial Sloan Kettering Cancer Center (MSK) is to assure that no caregiver of an MSK patient experiencing significant distress as a result of their caregiving responsibilities goes unidentified and deprived of necessary psychosocial services. This presentation will cover the steps taken and barriers faced in the development of the Clinic, including advocating for caregivers to receive their own unique medical records. Data regarding the number of caregivers seen for psychotherapy and for medication management will be presented, as will data regarding presenting complaints and average length of care. Also included is a discussion of the challenges faced in expanding and maintaining the capacity of the Clinic, especially in the setting of the pandemic during which caregivers' use of psychosocial care at MSK is notably higher than in years past. Several current adjunct approaches to address capacity needs currently being piloted will be discussed.

